# Antimicrobial Effects of Edible Mixed Herbal Extracts on Oral Microorganisms: An In Vitro Study

**DOI:** 10.3390/medicina59101771

**Published:** 2023-10-04

**Authors:** Se-Eun Yun, Byul-Bo ra Choi, Seoul-Hee Nam, Gyoo-Cheon Kim

**Affiliations:** 1 Corporate Affiliated Research Institute, Feagle Co., Ltd., Yangsan 50561, Republic of Korea; hoyalips@feagle.co.kr (S.-E.Y.); cbbrstar@feagle.co.kr (B.-B.r.C.); 2 Department of Dental Hygiene, Kangwon National University, Samcheok 25949, Republic of Korea; nshee@kangwon.ac.kr; 3 Department of Oral Anatomy, School of Dentistry, Pusan National University, Yangsan 50612, Republic of Korea

**Keywords:** *Acanthopanax sessiliflorus* Seem, antimicrobial, *Glycyrrhiza uralensis*, *Mentha piperascens*, oral pathogens, *Schizonepeta tenuifolia* Briq

## Abstract

*Background and Objectives*: The oral cavity is inhabited by pathogenic bacteria, whose growth can be inhibited by synthetic oral drugs, including antibiotics and other chemical compounds. Natural antimicrobial substances that elicit fewer negative side effects may serve as alternatives to synthetic agents for long-term use. Thus, the aim of this study was to evaluate the effects of edible mixed herbal extracts on the growth of oral pathogenic bacteria. *Materials and Methods*: The yield of each herbal extract was as follows: 5% *Schizonepeta tenuifolia* Briq (STB), 10.94% *Mentha piperascens* (MP), 5.47% *Acanthopanax sessiliflorus* Seem (AS), and 10.66% *Glycyrrhiza uralensis* (GU). The herbal extracts used included 0.5 mg/mL STB, 1.5 mg/mL MP, 1.5 mg/mL AS, and 2.0 mg/mL GU. Antimicrobial tests, morphological analyses (using scanning electron microscopy), microbial surface hydrophobicity measurements, and oral malodor reduction tests were performed using each extract. Statistical analyses were performed with IBM^®^ SPSS^®^ (version 24), using paired *t*-tests. *Results*: The mixed herbal extracts significantly inhibited the growth of *Streptococcus mutans, Enterococcus faecalis, Candida albicans,* and *Porphyromonas gingivalis* compared to the control (*p* < 0.001). Scanning electron microscopy results further revealed altered cellular morphology in the groups treated with the mixed herbal extracts. Additionally, the hydrophobicity assay results showed that the mixed herbal extracts reduced the oral adhesion capacities of bacteria (*p* < 0.001). Administration of the mixed herbal extracts also reduced the levels of volatile sulfur compounds, the main contributors to oral malodor (*p* < 0.001). *Conclusions*: Edible mixed herbal extracts can effectively eliminate oral pathogens and may be useful for improving oral health. The herbal extracts used were effective against all species of oral pathogens studied in this report.

## 1. Introduction

The human oral cavity is inhabited by over 700 different bacterial species, some of which are disease-causing pathogens that can induce gastrointestinal dysfunction and tooth loss [1]. White spot lesions that develop due to *Streptococcus mutans (S. mutans)* retention in plaque can accumulate around orthodontic brackets and lead to enamel demineralization, based on studies using simulated cariogenic environments [2]. When streptococci accumulate in the mouth (via attachment to the initially acquired pellicle), plaques (comprising the bacterial cells, glycoproteins, and polysaccharides) can form. This then generates acids, which form a solid cluster and cause demineralization [3,4]. Furthermore, oral pathogens also induce dental pulp disease and fungal infections [5], and can cause both oral and systemic diseases [6]. Thus, substances that can selectively inhibit dental bacteria are necessary to prevent diseases caused by oral microorganisms.

To prevent and/or suppress the progression of bacterial oral diseases, the causal pathogen must be eliminated; this is typically achieved with mouthrinses or antibiotics. Chlorhexidine gluconate (CHX) is a widely utilized agent in mouthwashes that is effective in preventing plaque formation and oral microbial growth [7]. Octenidine (OCT) significantly inhibited plaque formation, gingivitis, and oral microbial growth, and was more effective than placebos and other common chemical agents used for plaque control [7]. However, currently used antimicrobial agents such as CHX and OCT can exert harmful effects on the body after long-term use, as well as having various side effects, including microbial drug resistance [8,9]. 

To overcome these limitations, natural herbal extracts with high pharmacological efficacy and low toxicity have recently been applied to inhibit the growth of oral pathogens and prevent oral diseases [10,11]. For instance, flavanone and flavanol (isolated from natural herbal extracts) can inhibit the glucosyltransferases that cause dental caries [12], while natural mulberry [13], green tea [14], and cardinal [15] extracts can inhibit oral pathogens.

STB is an annual plant in the Lamiaceae family; the whole plant, commonly known as catnip, is primarily used for medicinal purposes due to its antioxidant, anti-inflammatory, antiviral [16], hemostatic, and antimicrobial [17] effects. MP is a perennial herb in the Lamiaceae family with known anticancer [18], anti-allergic [19], antioxidant [20], and analgesic effects [21]. AS is a deciduous shrub in the Araliaceae family, and its roots and bark (as well as those of plants in the same genus) are used as medicinal herbs. Chiisanoside is among the main components of these extracts and has anti-inflammatory, anti-diabetic, and antiviral effects [22], with positive efficacies in diabetes, cancer, and rheumatoid arthritis [23]. Chiisanoside also exerts potent antioxidant effects by preventing DNA damage caused by oxidative stress or protein glycation [24]. GU is a perennial herb in the Fabaceae family; its roots and rhizomes are used as herbal medicines with various pharmacological effects including detoxification, antiulcer, anti-inflammation, antispasmodic, anti-hepatotoxic, and antitussive activities [25]. Moreover, GU contains licopyranocoumarin and licoarylcoumarin, which have antiviral effects, as well as glycyrol and glycycoumarin, which have antimicrobial effects [26]. The antibacterial effects of mixtures of STB, MP, AS, and GU against oral pathogens are currently unclear. We aimed to determine whether edible natural extracts can be used as alternatives to overcome the limitations of conventional chemical mouthwashes. In this study, we aimed to confirm the antimicrobial (bactericidal) effect of these natural herbal extracts, and also evaluate whether they can mitigate halitosis in vitro. The null hypothesis was that no difference in survival rates would be observed between bacteria treated with and without the mixed herbal extracts.

## 2. Materials and Methods

### 2.1. Herbal Extracts

STB, MP, AS, and GU were purchased from Jecheon Herbal Medicine (Jecheon, Korea) and their antimicrobial efficacies were evaluated using antimicrobial tests. The herbs were dried and sectioned at the time of purchase. Each herbal material was finely ground, and 100 g of the ground material was mixed with 10× 70% ethanol (Sigma-Aldrich, St. Louis, MO, USA). The mixture was shaken at 60 °C for 12 h. The supernatants were isolated via filtration through filter paper and then concentrated using a vacuum evaporator (R-114; BÜUCHI, Flawil, Switzerland). The concentrate of each herbal extract was dehydrated using a freeze-dryer (FD, Ilshin Lab, Yangju, Korea) at −92 °C and then dissolved in dimethyl sulfoxide (Sigma-Aldrich, St. Louis, MO, USA) for subsequent use. The yield of each herbal extract was as follows: 5% STB, 10.94% MP, 5.47% ASS, and 10.66% GU [27]. Table 1 shows the concentration of each herbal extract, based on which combinations of STB, MP, ASS, and GU extracts were prepared.

### 2.2. Test Strains and Culture Conditions

Table 2 shows the microbial strains studied and their respective culture conditions. All strains were obtained from the Korean Collection for Type Cultures of the Korea Research Institute of Bioscience and Biotechnology (Daejeon, Korea). The strains were sub-cultured at least three times prior to use. The growth media were purchased from Sigma (Sigma-Aldrich, St. Louis, MO, USA).

### 2.3. Antimicrobial Efficacy of Mixed Herbal Extracts

To determine the viable cell count of the oral microbial strains, each test strain was cultured at 5 × 10^4^ colony-forming units (CFU)/mL; a mixture of 100 μL of bacteria and 900 μL of the mixed herbal extracts was prepared and cultured for 6 h. The cultured bacteria were spread on an agar dish (SPL Life Sciences, Pocheon, Korea) and cultured for 24 h in a 37 °C incubator (SPL Life Sciences, Pocheon, Korea), after which the viable cells were counted [28]. To improve the statistical validity, each measurement was performed in triplicate. The rate of microbial growth inhibition was calculated using the following equation:

$$Rate\;of\;inhibition\;\left(\%\right)=\frac{Count\;of\;control\;group-Count\;of\;experimental\;group}{Count\;of\;control\;group}\times 100$$

### 2.4. Morphological Examination of Oral Pathogens via Scanning Electron Microscopy

Bacteria in the control and experimental groups were inoculated in liquid media at 5 × 10^5^ CFU/mL. In the experimental group, the mixed herbal extracts were diluted to 5.5 mg/mL and added to the liquid media. The control and experimental groups were cultured at 37 °C for 24 h, after which the bacteria were transferred to a 1.5 mL tube for centrifugation at 3000 revolutions per minute (rpm) for 5 min. The supernatant was removed, and the pellet was washed with distilled water and centrifuged again at 5000 rpm for 5 min. The supernatant was removed, and the pellet was fixed in 2.5% glutaraldehyde solution (0.1 M sodium cacodylate buffer, pH 7.2, 4 °C) for 1 h, before being centrifuged again. Subsequently, 300 μL of 1% osmium tetroxide was added to the supernatant; a drop of the mixture was then placed on a cover slide for fixation for 1 h. The fixed bacteria were dehydrated by successively increasing the ethanol concentration to 50%, 70%, 80%, 95%, and 100%. After freeze-drying and gold-coating, images of each sample were acquired using scanning electron microscopy (Hitachi, Tokyo, Japan) [29].

### 2.5. Microbial Surface Hydrophobicity

The effects of the mixed herbal extracts on the surface hydrophobicity of each microbial strain were measured. The absorbance of each strain was set to 0.3 for optical density (OD)_550_ analysis. A total of 2 mL of the microbial suspension was added to a 15 mL round tube along with an equal amount of n-hexadecane (Sigma-Aldrich, St. Louis, MO, USA), and the sample was mixed for 1 min with a vortex mixer. The mixed herbal extracts were left to stand at room temperature for 30 min, after which the supernatant was removed and the absorbance at OD_550_ was measured using a spectrophotometer (Multiskan Go, Thermo Fisher Scientific, Waltham, MA, USA). Cell surface hydrophobicity was calculated using the following equation: %HP (hydrophobic) = [OD (initial) − OD (expt)] × 100/OD (initial). All measurements were performed in triplicate for statistical analysis [30].

### 2.6. Oral Malodor Reduction

Oral chroma (CHM-1, Nissha FIS, Inc., Osaka, Japan) was used to measure reductions in oral malodor. For the control group, liquid medium was placed in a 15 mL tube and inoculated with 5 × 10^5^ CFU/mL bacteria. The experimental group was prepared in the same manner as the control group; however, 5.5 mg/mL of mixed herbal extracts was also added. The control and experimental groups were cultured for 6 h. A syringe was then inserted into the tube to collect the gas from the cultured bacteria. The gas was applied to the oral chroma, and the volatile sulfur compound (VSC) concentrations were measured. All measurements were performed in triplicate [31].

### 2.7. Data Analysis

All experiments were repeated three times, and the results were analyzed using SPSS Statistics 24.0 software (SPSS, Inc., Chicago, IL, USA) [32]. The normality of the data was assessed using the Shapiro–Wilk test. The significance of differences between the control and experimental groups was determined using paired *t*-tests. *p* < 0.05 was considered statistically significant.

## 3. Results

### 3.1. Inhibitory Effects of Mixed Herbal Extracts on Oral Pathogens

**Figure 1:**
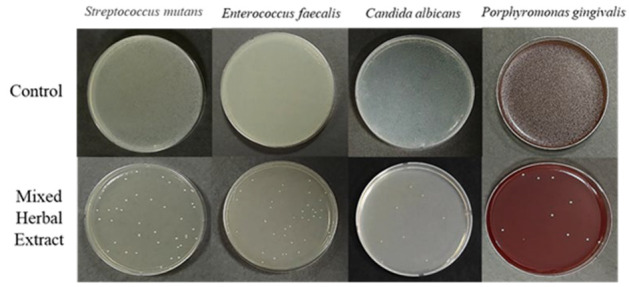
Colony-forming units (CFU) of oral pathogens following treatment with mixed herbal extracts (containing *Schizonepeta tenuifolia*, *Mentha piperascens, Acanthopanax sessiliflorus*, and *Glycyrrhiza uralensis*). Each white dot on the plates indicates an oral pathogen colony.

We aimed to determine the antimicrobial effects of the mixed herbal extracts (containing STB, MP, AS, and GU) on each oral pathogen strain. Treatment with the mixed herbal extracts significantly decreased the colony-forming ability of *S. mutans, Enterococcus faecalis (E. faecalis), Candida albicans (C. albicans)*, and *Porphyromonas gingivalis* (Figure 1; *p* < 0.001). When treated with the mixed herbal extracts, *S. mutans* decreased from 5.90 ± 0.01 to 1.48 ± 0.03**, *E. faecalis* decreased from 5.95 ± 0.01 to 1.49 ± 0.05**, *C. albicans* decreased from 5.85 ± 0.02 to 0.90 ± 0.03**, and *P. gingivalis* decreased from 5.74 ± 0.01 to 0.95 ± 0.03**. The counts of *S. mutans, E. faecalis, C. albicans*, and *P. gingivalis* were reduced by 3.94 logarithm (log), 4.01 log, 4.94 log, and 4.79 log, respectively, when treated with the mixed herbal extracts (Table 3).

### 3.2. Morphological Examination of Oral Pathogens

**Figure 2:**
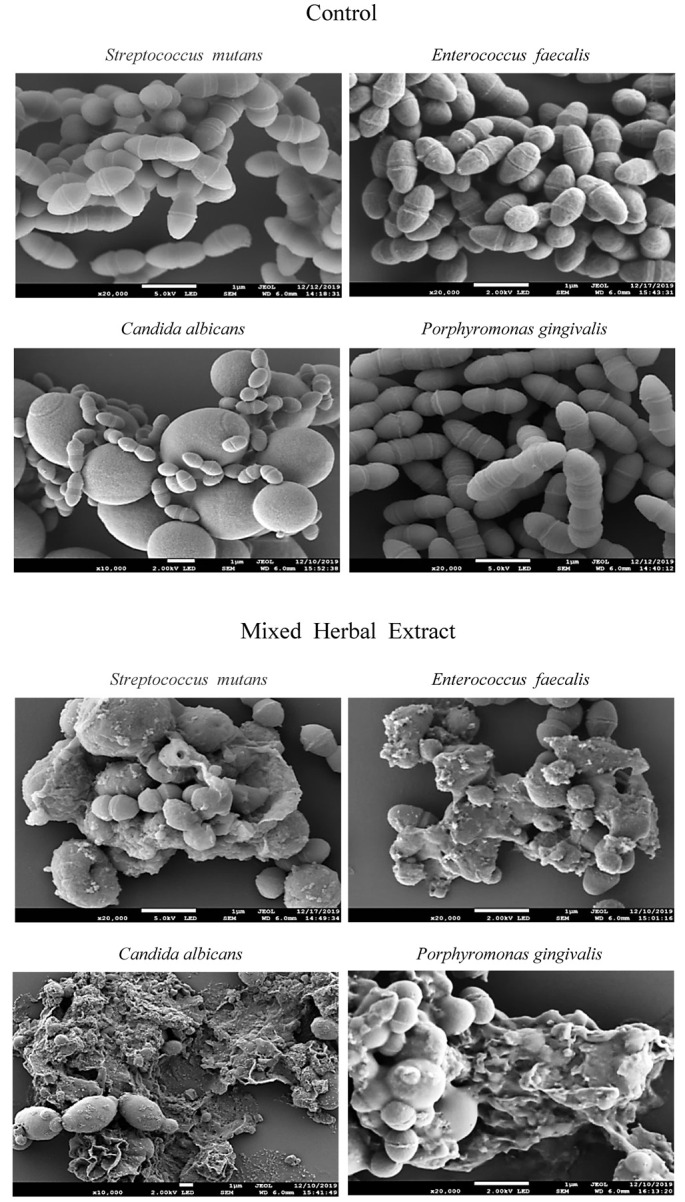
Scanning electron microscope images of studied microorganisms.

The cell surfaces of each bacterial test strain were examined using scanning electron microscopy after being treated with or without the mixed herbal extracts (Figure 2). All strains cultured with the mixed herbal extracts exhibited visible damage, with altered cellular morphologies due to the destruction of cell walls and membranes. Non-treated strains displayed normal cellular morphologies.

### 3.3. Bacterial Surface Hydrophobicity

The surface hydrophobicities of each test strain are shown in Table 4. When compared to the control, bacteria treated with the mixed herbal extracts exhibited significantly decreased hydrophobicity with increasing treatment time (*p* < 0.05). Similar results were observed for *E. faecalis*, *C. albicans*, and *P. gingivalis*. The relative hydrophobicity of *S. mutans* (assuming 100 in the untreated group) decreased to 61.20 ± 3.20**, 60.37 ± 0.73*, 59.23 ± 1.73*, and 57.35 ± 1.47%* after being treated with the mixed herbal extract for 1, 3, 5, and 10 min, respectively. The relative hydrophobicity of *E. faecalis* decreased to 85.63 ± 3.70*, 83.10 ± 1.46*, 74.34 ± 2.67*, and 72.06 ± 1.10%*, respectively. The relative hydrophobicity of *C. albicans* decreased to 80.89 ± 1.17*, 38.75 ± 2.89*, 27.91 ± 6.52*, and 19.91 ± 2.73%*, respectively. The relative hydrophobicity of *P. gingivalis* decreased to 44.11 ± 2.10*, 30.78 ± 2.13*, 26.96 ± 1.39**, and 22.14 ± 1.50%*, respectively.

After 10 min of treatment with the mixed herbal extracts, the surface hydrophobicity decreased by 42.65% in *S. mutans*, 27.94% in *E. faecalis*, 80.08% in *C. albicans*, and 77.86% in *P. gingivalis*.

### 3.4. Measurement of VSCs

The effects of the mixed herbal extracts on oral malodor were determined by measuring the generated VSCs, which are the main components of oral malodor (Table 5). Treatment with the mixed herbal extracts led to significant decreases in the relative production of VSCs; VSC production decreased by 91.25% in *S. mutans*, 97.03% in *E. faecalis*, 88.99% in *C. albicans*, and 100% in *P. gingivalis* when compared with the control group (*p* < 0.001).

## 4. Discussion

Various microbial species inhabit the oral cavity, and the growth of pathogenic bacteria can cause oral diseases. Frequent consumption of refined carbohydrates accelerates the demineralization of tooth enamel due to dental caries, causing imbalances in remineralization and demineralization [33].

*S. mutans* refers to a group of seven closely related species that are collectively known as mutans Streptococci. *S. mutans* are commonly found in the mouth, pharynx, and intestines. The etiology of dental caries is heavily influenced by *S. mutans* and *Streptococcus sobrinus* [34]. Oral diseases such as caries, endodontic infections, periodontitis, and peri-implantitis have also been linked to *E. faecalis*, the predominant human Enterococcus. *E. faecalis* has been observed to comprise 3.7–35% of the oral microbiota in periodontitis patients [35]. *Candida albicans* is a commensal fungal species that commonly colonizes human mucosal surfaces. *C. albicans* has been observed in carious dentin/dentine tubules [36]. *P. gingivalis* is the major causative agent of periodontitis, a chronic inflammatory disease that causes tooth loss and degeneration of the gingiva, alveolar bones, and periodontal ligaments [37].

Oral diseases such as dental caries, periodontal disease, periapical lesions, and oral candidiasis are the main causes of tooth loss and oral malodor, for which effective management methods are urgently needed. Mouthwashes containing chemical agents are commonly used to eliminate bacteria and/or inhibit microbial activity. In a previous study, the use of mouthwash containing antimicrobial and anticaries agents decreased dental plaque formation by approximately 61% [38]. In other studies, mouthwashes containing eucalyptol and thymol or cetylpyridinium chloride were able to inhibit dental plaque formation and gingivitis, and both exhibited excellent antimicrobial activity against oral pathogens [39,40]. However, long-term use of mouthwash can also cause discoloration of the teeth and/or soft tissues and affect taste sensation [41]. In addition, the chemical agents in mouthwash can affect the normal microbial flora along with pathogens in the oral cavity. Furthermore, ethanol-containing mouthwashes may increase the risk of oral cancer and thus should only be used for limited periods [42]. Recent studies have focused on identifying natural substances as alternatives for synthetic chemical agents to selectively eliminate pathogenic bacteria.

In this study, the effects of a natural herbal extract mixture (containing STB, MP, AS, and GU) on oral bacteria were determined to help develop a natural-substance-based mouthwash. All herbal extracts used in the experiment are edible; thus, the proposed product can be gargled or can be safely consumed. In particular, STB [43] and GU [44] have been reported to inhibit the growth of *Helicobacter pylori*, a major cause of gastric cancer and gastritis. The herbs used in our proposed mixture exhibit anti-inflammatory and antimicrobial effects; however, excessive doses of GU can elicit negative effects such as severe hypertension, hypokalemia, and hypermetabolism. Hence, GU must be used with caution [45]. Approximately 20% of GU is comprised of glycyrrhizin [46], which possesses mineralocorticoid activity to degrade nicotine and alcohol, thereby acting as a detoxifying agent [45]. The recommended daily dose of GU for adults is 0.7 g, although a dose of ≥0.1 g/mL can have negative effects in patients with neurological disease [47]. Accordingly, when preparing the mixed herbal extracts, only a small amount of the raw GU material was added to minimize the risks of side effects. 

Our experimental results showed that *S. mutans*, which causes dental caries, and *E. faecalis*, which is a causative agent of dental pulp disease, were reduced by ≥3 log CFU/mL under treatment with the mixed herbal extracts. Additionally, the numbers of *C. albicans*, a fungal strain that infects the oral mucosa, and *P. gingivalis*, which causes periodontal disease, were reduced by ≥4 log CFU/mL; thus, our proposed mixture has significant efficacy in inhibiting oral pathogens. Based on these findings, the null hypothesis was rejected. These results indicate that the mixed herbal extracts had stronger effects on Gram-positive fungi and Gram-negative bacteria than on Gram-positive bacteria. This disagrees with the findings of a previous study (Koohsari et al.), which found that the extract was more effective against Gram-positive bacteria than Gram-negative bacteria [48].

Morphological examination of each microbial strain showed that the oral microorganisms exhibited disorganized damage after treatment with the mixed herbal extracts. Particularly, *C. albicans* showed an overall damaged morphology. Similarly, Sangetha et al. [49] treated *C. albicans* with *Cassia spectabilis* leaf extract for 24 h and found that the extract damaged the microbial surface. We obtained identical results after 24 h of treatment with the mixed herbal extracts, which further validates that this herbal extract mixture possesses antimicrobial activity and can induce morphological damage in oral pathogens.

The hydrophobicity of cells is closely associated with microbial adhesion, and microbial strains with a high level of hydrophobicity can form biofilms. Many studies have reported that chlorohexidine, which is widely used as a component of mouthwashes, induces microbial adhesion. However, in one study, treatment of *S. mutans* with a high concentration of chlorohexidine decreased microbial adhesion, whereas in another study, biofilm formation was increased [50,51]. We found that treatment with the mixed herbal extracts reduced microbial adhesion, indicating that these extracts reduced the surface hydrophobicity of oral pathogens. Further studies are needed to investigate the mechanisms underlying the effects of these mixed herbal extracts on microbial cell hydrophobicity.

Oral malodor, also known as halitosis, is a disease that can significantly impact quality of life. It can be caused by oral, physiological, and/or pathological factors, with oral factors considered to be the main cause [52]. The main compounds contributing to bacteria-induced oral malodor are VSCs such as hydrogen sulfide, methyl mercaptan, and dimethyl sulfide. Indeed, VSC levels increase in patients with oral diseases such as dental caries [53]. Several studies have reported that reducing the levels of anaerobic oral bacteria resulted in decreases in VSC levels [54,55]. We demonstrated that the mixed herbal extracts reduced oral malodor by suppressing VSC formation. Treatment with the mixed herbal extracts inhibited the growth of *P. gingivalis* (the causal bacteria of periodontal disease) by 100%, indicating that the extracts have the potential to reduce oral malodor. We verified the antimicrobial effects of the mixed herbal extracts on oral disease-causing strains.

Based on these results and the mitigation of oral malodor, edible mixed herbal extracts may represent an effective antimicrobial agent against oral disease pathogens with minimal side effects since they are naturally derived. Thus, edible mixed herbal extracts are expected to have a strong antibacterial effect that can replace the activities of CHX and OCT in existing mouthwash and have a high potential for practical use.

However, as mixed extracts were used, the specific component(s) responsible for the observed antimicrobial effects remains unclear. Thus, further studies are needed to isolate and examine the individual effects of the edible mixed herbal extracts’ components. Therefore, it is necessary to conduct further research to confirm the antibacterial effects of each component of the extract against oral bacteria. Finally, our findings on the efficacy of edible mixed herbal extracts will provide guidance for clinical decision making in the future, as these compounds are tested via clinical trials.

## 5. Conclusions

We examined the antimicrobial effects of mixed herbal extracts on oral pathogens and found that the abundances of the four evaluated strains of oral pathogens were reduced after treatment. Additionally, treatment with the mixed herbal extracts altered the morphologies of *S. mutans*, *E. faecalis*, *C. albicans*, and *P. gingivalis* cells based on scanning electron microscopy observations. All the studied oral pathogen strains also showed decreased hydrophobicity after treatment, and *C. albicans* exhibited the greatest decrease. Treatment with the mixed herbal extracts also reduced malodor caused by these pathogens. In particular, the levels of *P. gingivalis*-related VSCs were reduced by 100%. Based on our results, it can be concluded that the proposed natural mixed herbal extracts (containing STB, MP, AS, and GU) may demonstrate efficacies in inhibiting and/or limiting bacterial and fungal growth and preventing halitosis. However, further toxicity tests using human oral cavity cells are needed to confirm their safety.

## Figures and Tables

**Table 1:** Composition of mixed herbal extracts used in the experiments.

Scientific Name	Plant Part	Concentration (mg/mL)
*Schizonepeta tenuifolia*	Above-ground	0.5
*Mentha piperascens*	Leaf	1.5
*Acanthopanax sessiliflorus* Seem	Bark	1.5
*Glycyrrhiza uralensis*	Root	2

**Table 2:** Studied microorganisms and culture requirements.

Strains	Straining Property	Culture Requirement	Media
*S. mutans* ^a^ (KCTC ^b^3065)	Gram-positive	Facultative anaerobic	Brain heart infusion/agar
*E. faecalis* ^c^ (KCTC2011)	Gram-positive	Facultative anaerobic	Brain heart infusion/agar
*C. albicans* ^d^ (KCTC7965)	Gram-positive	Aerobic	YM broth/agar
*P. gingivalis* ^e^ (KCTC5352)	Gram-negative	Anaerobic	Tryptic soy broth/agar

^a^ *Streptococcus mutans* (*S. mutans*); ^b^ Korean Collection for Type Cultures (KCTC); ^c^ *Enterococcus faecalis* (*E. faecalis*); ^d^ *Candida albicans* (*C. albicans*); ^e^ *Porphyromonas gingivalis* (*P. gingivalis*).

**Table 3:** Growth inhibition of four microorganisms treated with mixed herbal extracts.

Group	*S. mutans* ^a^	*E. faecalis* ^b^	*C. albicans* ^c^	*P. gingivalis* ^d^
Control	5.90 ± 0.01	5.95 ± 0.01	5.85 ± 0.02	5.74 ± 0.01
Mixed herbal extracts	1.48 ± 0.03 **	1.49 ± 0.05 **	0.90 ± 0.03 **	0.95 ± 0.03 **

** *p* < 0.001; ^a^ *Streptococcus mutans* (*S. mutans*); ^b^ *Enterococcus faecalis* (*E. faecalis*); ^c^ *Candida albicans* (*C. albicans*); ^d^ *Porphyromonas gingivalis* (*P. gingivalis*).

**Table 4:** Effects of mixed herbal extracts on the hydrophobic properties of the studied microorganisms.

Group	*S. mutans* ^a^	*E. faecalis* ^b^	*C. albicans* ^c^	*P. gingivalis* ^d^
Control	100	100	100	100
1 min ^e^	61.20 ± 3.20 **	85.63 ± 3.70 *	80.89 ±1.17 *	44.11 ± 2.10 *
3 min ^e^	60.37 ± 0.73 *	83.10 ± 1.46 *	38.75 ± 2.89 *	30.78 ± 2.13 *
5 min ^e^	59.23 ± 1.73 *	74.34 ± 2.67 *	27.91 ± 6.52 *	26.96 ± 1.39 **
10 min ^e^	57.35 ± 1.47 *	72.06 ± 1.10 *	19.91 ± 2.73 **	22.14 ± 1.50 **

* *p* < 0.05, ** *p* < 0.001; ^a^ *Streptococcus mutans* (*S. mutans*); ^b^ *Enterococcus faecalis* (*E. faecalis*); ^c^ *Candida albicans* (*C. albicans*); ^d^ *Porphyromonas gingivalis* (*P. gingivalis*); ^e^ minute (min).

**Table 5:** Effects of mixed herbal extracts on the production of volatile sulfur compounds.

Group	*S. mutans* ^a^	*E. faecalis* ^b^	*C. albicans* ^c^	*P. gingivalis* ^d^
Control	1244.33 ± 22.24	109.00 ± 20.60 **	644.00 ± 28.00	47.00 ± 13.01
Mixed herbal extracts	109.00 ± 20.60 **	38.33 ± 10.80 **	71.00 ± 12.53 **	0.00 ± 0.00 **

** *p* < 0.001; ^a^ *Streptococcus mutans* (*S. mutans*); ^b^ *Enterococcus faecalis* (*E. faecalis*); ^c^ *Candida albicans* (*C. albicans*); ^d^ *Porphyromonas gingivalis* (*P. gingivalis*).

## Data Availability

The data presented in this study are available upon request from the corresponding author.
